# Fluorogenic Peptide Substrate for Quantification of Bacterial Enzyme Activities

**DOI:** 10.1038/srep44321

**Published:** 2017-03-13

**Authors:** Ismail H. Al-Abdullah, Karine Bagramyan, Shiela Bilbao, Meirigeng Qi, Markus Kalkum

**Affiliations:** 1 Department of Translational Research and Cellular Therapeutics, Diabetes and Metabolism Research Institute, Beckman Research Institute of the City of Hope, USA; 2 Department of Molecular Immunology, Beckman Research Institute of the City of Hope, USA.

## Abstract

A novel peptide substrate (A G G P L G P P G P G G) was developed for quantifying the activities of bacterial enzymes using a highly sensitive Fluorescence Resonance Energy Transfer (FRET) based assay. The peptide substrate was cleaved by collagenase class I, II, Liberase MTF C/T, collagenase NB1, and thermolysin/neutral protease, which was significantly enhanced in the presence of CaCl_2_. However, the activities of these enzymes were significantly decreased in the presence of ZnSO_4_ or ZnCl_2_. Collagenase I, II, Liberase MTF C/T, thermolysin/neutral protease share similar cleavage sites, L^↓^G and P^↓^G. However, collagenase NB1 cleaves the peptide substrate at G^↓^P and P^↓^L, in addition to P^↓^G. The enzyme activity is pH dependent, within a range of 6.8 to 7.5, but was significantly diminished at pH 8.0. Interestingly, the peptide substrate was not cleaved by endogenous pancreatic protease such as trypsin, chymotrypsin, and elastase. In conclusion, the novel peptide substrate is collagenase, thermolysin/neutral protease specific and can be applied to quantify enzyme activities from different microbes. Furthermore, the assay can be used for fine-tuning reaction mixtures of various agents to enhance the overall activity of a cocktail of multiple enzymes and achieve optimal organ/tissue digestion, while protecting the integrity of the target cells.

Clinical islet transplantation has proven to be an effective therapeutic method to treat type 1 diabetes mellitus and to improve glycemic control^1^,^2^,^3^. Successful islet transplantation depends largely on isolating sufficient islet mass from suitable pancreata^4^. The essential procedure for isolating islets is to digest pancreatic tissue and free the islets from the abundant extracellular matrix (ECM) components^5^. It has been reported that the human pancreas contains multiple extracellular matrixes (ECM) including: collagen types I, III, IV, laminin, elastin, and fibronectin^5^,^6^,^7^. An Adult pancreas has an abundance of collagen bands and thus isolating islets has been a difficult and challenging task for obtaining maximum islet yields^8^. Collagenase is the main component of the enzyme cocktail that is currently being used to digest the pancreas to free the islets. It has been reported that collagenase contains class I (60%) and II (40%) isoforms^9^. Highly purified enzymes with low endotoxin levels were developed by Roche Liberase MTF C/T kit containing collagenase (I + II) and thermolysin, and by Serva collagenase NB1 and neutral protease. Despite the effort to standardize the enzymatic procedure and manufacture a reliable GMP grade enzyme for pancreatic islet isolation^10^, the optimal enzymes to digest the pancreas to achieve successful islet isolation is still remaining a challenge. Hence, many centers use their own combination of digestive enzymes, which are usually based on the experience of their respective islet isolation teams^11^. For successful digestion of the extracellular matrix, thermolysin/neutral protease is also added to the collagenase to synergistically degrade various collagen bands. Collagenase and neutral protease are produced from *Clostridium histolyticum*^12^, and thermolysin is purified from *Bacillus thermoproteolyticus rokko*^13^. Recently, a study conducted on 400 human pancreata has shown that an appropriate ratio of collagenase class I and II is critical to collectively enhance the activity and potency of enzymatic digestion for maximum islet yields^14^. The data clearly shows that rapid digestion of the organ is essential to protect the quality of the isolated islets^14^. It is conceivable that the presence of neutral protease within collagenase may result in the degradation of the enzyme, thus affecting the activity. This may ultimately lead to inconsistent results^15^.

In addition to the inconsistency in enzyme potency, another important limitation in the field of islet isolation is the inevitability of using donor pancreas to test the efficacy of enzyme cocktails. On one hand, this approach is costly; on the other hand, the variability in donor characteristics per se introduces more variables that affect enzyme evaluation. Currently, there are several methods available to measure collagenase activity^9^,^16^. In particular, the Wünsch assay has been used to measure class II collagenase^16^, but was found to be unsuitable for measuring class I collagenase activity. These measurements are carried out using each manufacture’s own protocol, which make it difficult to standardize the assay. Furthermore, the cleavage sites of pancreas ECM by varies enzymes have not been investigated before, since this will help to further refine the characteristics of enzyme(s) used for pancreas digestion. Hence, a more accurate and reliable method is needed to quantify the enzyme activity. In this study, we first synthesized and characterized a novel peptide substrate that can be used to assess the collagenolytic activity of the enzymes including collagenase class I, II and thermolysin/neutral protease using Fluorescence Resonance Energy Transfer (FRET). The method is sensitive to determine the kinetic parameters of various enzymes using micromolar concentrations of substrate^17^,^18^,^19^,^20^,^21^. The substrate contains 5-CarboxyFluorescein-Aminohexyl Amidite (5-Fam) fluorogenic groups. The principle of the assay is based on the fact the donor fluorophore and the acceptor fluorophore (quencher) are in sufficient proximity in the substrate to allow a resonance energy transfer between the fluorophore and the quencher. The change in resonance energy transfer and increase in fluorescence intensity upon substrate cleavage are associated with higher intensity of enzymatic reaction. Furthermore, we examined how the enzymes cleave the peptide substrate under different conditions that exist in actual process of pancreatic tissue digestion: (1) effect of reaction components on enzyme activity; (2) effect of pH; and (3) effect of endogenous pancreatic proteases on the peptide substrate.

## Results

### Characterization of synthesized peptides

The pancreas has been used as a surrogate for enzyme evaluation. Although, this has been relatively helpful in understanding pancreas digestion and ultimately freeing islets, the procedure is expensive, lengthy, and challenging. Hence, the need for a simple and effective assay to evaluate and standardize enzyme activity is highly desirable to overcome these problems^11^. The unique characteristic of the enzyme substrate developed is described in Table 1. Our new substrate is 5Fam-AGGPLGPPGPGGK-dabcyl, which has different donor/acceptor fluorophores group localization, which can be cleaved by thermolysin as compared to a previous study^18^. Complete amino-acid sequences of the ECM: collagens (I, II, III, IV, V), laminin, and fibronectin were obtained in FASTA format from UniProtKB^22^. The amino-acid sequence of our newly synthesized peptide substrate was compared to the ECM using BLAST (basic local alignment search tool)^23^. Interestingly, our peptide has a uniquely similar sequence to collagen bands (I, II, III, IV, V) with identities of 80%, 80%, 80%, 90%, and 80%, respectively, as shown in Table 1.

**Figure 1:**
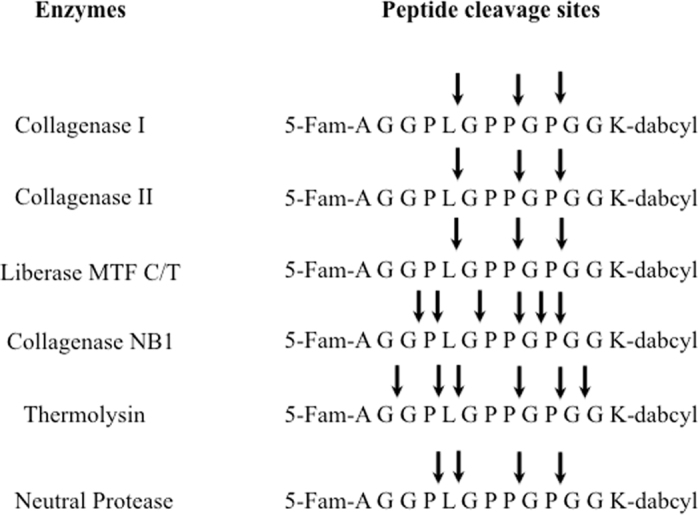
Cleavage sites on peptide substrate when react with different enzymes.

In our study, the cleavage site was determined based on the results of mass spectrometric analysis (Supplementary Information) of collagenase peptide fragments obtained from an enzymatic reaction with various enzymes tested. As noted in Fig. 1, the results show that Roche collagenase class I and II have similar cleavage sites on the peptide substrate, L^↓^G, and P^↓^G. However, Serva collagenase NB1 has three different cleavage sites: G^↓^P, P^↓^L, and P^↓^G. The neutral protease, which is produced by *Clostridium histolysticum*, has three cleavage sites: P^↓^L, L^↓^G, and P^↓^G, while thermolysin prepared from *Bacillus thermoproteolyticus rokko* has four cleavage sites: P^↓^L, L^↓^G, P^↓^G, and G^↓^G. Interestingly, beside collagenase NB1, all other enzymes share the same cleavage sites, P^↓^G and L^↓^G.

### Effect of CaCl_2_ on enzyme activity

**Figure 2:**
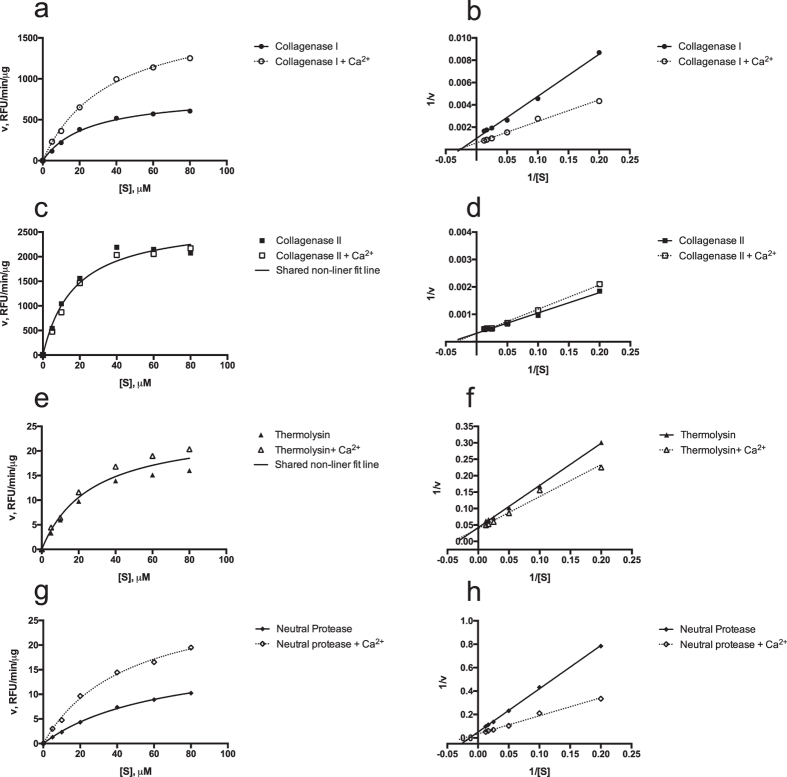
Plots of Michaelis-Menten (**a**,**c**,**e**,**g**) and Lineweaver-Burk (**b**,**c**,**f**,**h**) double-reciprocal plots analysis of enzyme cleavage of peptide substrate. The substrate was used at various concentrations (5, 10, 20, 40, 60, and 80 μM) in the presence of collagenase I (0.45 μg/ml), neutral protease (20 μg/ml), collagenase II (0.3 μg/ml), and thermolysin (17 μg/ml). The reaction of 0.45 μg/ml of CI (n = 3, (**a**,**b**) and 20 μg/ml of NP (n = 3, (**g**,**h**) were significantly enhanced in the presence of CaCl_2_ (CI, p < 0.0001; NP, p < 0.0001). The reaction curves of both 0.3 μg/ml of CII (n = 3, (**c**,**d**) and 17 μg/ml thermolysin (n = 3, (**e**,**f**) did not change significantly when CaCl_2_ was added (CII, p = 0.490; thermolysin, p = 0.239), which is justified by the occurrence of shared non-liner fit line between the conditions of presence and absent of CaCl_2_ (**c**,**e**).

The influence of CaCl_2_ on enzyme activities using the peptide substrate was tested since this chemical is commonly used during pancreatic islet isolation. Figure 2 shows that the enzyme kinetics increasing the relative fluorescent units (RFU) in a substrate-dependent manner. The reaction of 0.45 μg/ml collagenase I (n = 3, Fig. 2a,b) was significantly enhanced in the presence of CaCl_2,_ indicated by kinetic parameters V_max_ (without vs with Ca^2+^: 808.8 ± 59.5 vs 1845.0 ± 226.0 RFU/min/μg; p < 0.0001) and K_m_ (without vs with Ca^2+^: 24.7 ± 4.8 vs 36.7 ± 10.2 μM; p < 0.0001) (Table 2). So was the reaction of 20 μg/ml neutral protease (n = 3, Fig. 2g,h), showed by V_max_ (without vs with Ca^2+^: 18.8 ± 3.9 vs 30.3 ± 11.0 RFU/min/μg; p < 0.0001) and K_m_ (without vs with Ca^2+^: 65.6 ± 24.5 vs 46.0 ± 34.9 μM; p < 0.0001) (Table 2).

However, as shown in Fig. 2c,d,e,f, the reaction curves of both 0.3 μg/ml of collagenase II (n = 3, Fig. 2c,d) and 17 μg/ml of thermolysin (n = 3, Fig. 2e,f) did not significantly change when CaCl_2_ was added. There were no significantly differences in terms of kinetic parameters V_max_ and K_m_ (collagenase II, p = 0.490; thermolysin, p = 0.239). For collagenase I and neutral protease, the significant enhancement of reaction in the presence of CaCl_2_ was indicated by two distinctive non-liner fit lines (Fig. 2a,g). For collagenase II and thermolysin, no significant differences in terms of kinetic parameters was reflected by the occurrence of shared non-liner fit line between the conditions of presence and absent of CaCl_2_ (Fig. 2c,e).

### Effect of zinc ion on enzyme activity

**Figure 3:**
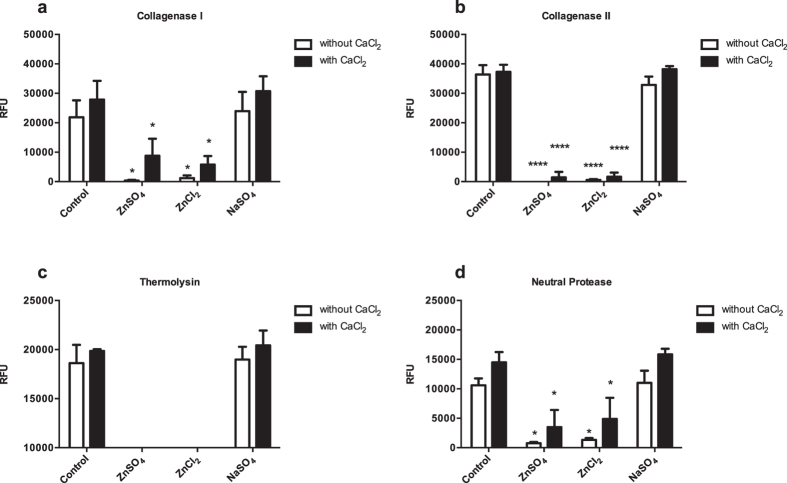
Effect of zinc on enzyme activity. The enzyme tested were collagenase I (**a**), collagenase II (**b**), thermolysin (**c**), and neutral protease (**d**). The substrate used in this experiment was at a concentration of 20 μM (n = 3). The values were expressed as mean ± standard error of mean (SEM). *p < 0.05, **p < 0.01, and ****p < 0.0001 compared to control. The undetectable RFU values were expressed by 0.

This experiment was conducted since the chemical containing zinc ions are supplemented in culture media used for islet culture post isolation. The effect of zinc ions on enzyme activity was investigated using ZnSO_4_ (16.7 μM) and ZnCl_2_ (16.7 μM) solution. NaSO_4_ (16.7 μM) was used as a control to compare the Zn^2+^ effect to the influence of Na^+^ or SO_4_^2−^ ions. The reaction was conducted in the presence and absence of CaCl_2_ (4.14 mM). This concentration was found to be optimal for pancreas digestion^11^. The substrate used in this experiment was at a concentration of 20 μM. As shown in Fig. 3, for all the four enzymes tested, the measured RFU was significantly decreased by adding either ZnSO_4_ or ZnCl_2,_ regardless of presence or absence of CaCl_2_, whereas, no significant difference was found by adding NaSO_4._ i) The RFU values for collagenase I are: in the presence of CaCl_2_, 27833 ± 6377 (control), 8788 ± 5782 (ZnSO_4_), 5776 ± 2904 (ZnCl_2_), and 30733 ± 5033 (NaSO_4_); in the absence of CaCl_2_, 21850 ± 5749 (control), 347 ± 261 (ZnSO_4_), 1201 ± 895 (ZnCl_2_), and 23933 ± 6549 (NaSO_4_). ii) The RFU values for collagenase II are: in the presence of CaCl_2_, 37250 ± 2451 (control), 1456 ± 1855 (ZnSO_4_), 1652 ± 1415 (ZnCl_2_), and 38183 ± 1027 (NaSO_4_); in the absence of CaCl_2_, 36383 ± 3188 (control), 0 (ZnSO_4_), 583 ± 274 (ZnCl_2_), and 32850 ± 2817 (NaSO_4_). iii) The RFU values for thermolysin are: in the presence of CaCl_2_, 19850 ± 177 (control), 0 (ZnSO_4_), 0 (ZnCl_2_), and 20433 ± 1509 (NaSO_4_); in the absence of CaCl_2_, 18616 ± 1867 (control), 360 ± 545 (ZnSO_4_), 205 ± 63 (ZnCl_2_), and 18983 ± 1303 (NaSO_4_). iv) The RFU values for neutral protease are: in the presence of CaCl_2_, 14483 ± 1761 (control), 3488 ± 2909 (ZnSO_4_), 4887 ± 3576 (ZnCl_2_), and 15850 ± 956 (NaSO_4_); in the absence of CaCl_2_, 10600 ± 1156 (control), 768 ± 192 (ZnSO_4_), 1330 ± 312 (ZnCl_2_), and 11005 ± 2072 (NaSO_4_). The undetectable RFU values were expressed by 0.

### Effect of EGTA on enzyme activity

**Figure 4:**
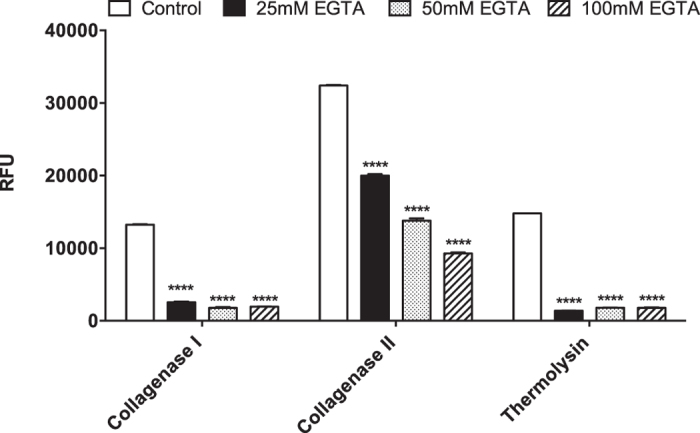
Effects of chelating agent EGTA on enzymatic activity. There were significant differences of RFU levels between the controls and varies concentrations of EGTA (25, 50, and 100 mM) for collagenase I (0.45 μg/ml), collagenase II (0.3 μg/ml), and thermolysin (17 μg/ml) (****p < 0.0001). The assay was conducted using a substrate concentration of 20 μM (n = 2).

Increasing concentrations of EGTA (25 mM, 50 mM, and 100 mM) were used to study the effect of chelating agents on the enzyme activity. The results were shown in Fig. 4. The RFU values for collagenase I are: 13250 ± 50 (control), 2540 ± 110 (25 mM), 1780 ± 120 (50 mM), and 1950 ± 10 (100 mM). The values for collagenase II are: 32400 ± 100 (control), 20000 ± 200 (25 mM), 13800 ± 300 (50 mM), and 9280 ± 140 (100 mM). The values for thermolysin are: 14800 ± 0 (control), 1395 ± 25 (25 mM), 1805 ± 35 (50 mM), and 1790 ± 60 (100 mM). There were significant differences of RFU levels between the controls and various concentrations of EGTA (25, 50, and 100 mM) for collagenase I (0.45 μg/ml), collagenase II (0.3 μg/ml), and thermolysin (17 μg/ml) (Fig. 4, p < 0.0001). EGTA is a mono calcium-chelating agent and therefore the presence of EGTA chelated Ca^2+^ further substantiates the importance of CaCl_2_ during digestion process.

### Effect of pH on enzyme activity

**Figure 5:**
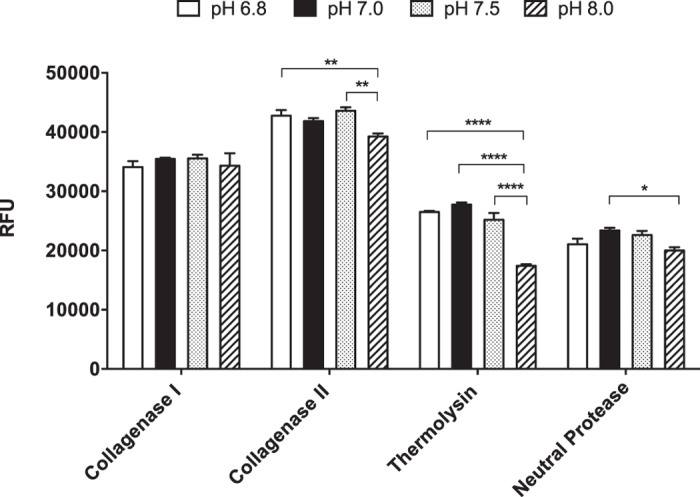
Effects of pH on enzymatic activity. All the enzymes except collagenase I, there were significant differences between different levels of pH. For collagenase II, the reaction was significantly diminished at pH 8.0, compared with pH 6.8 and pH 7.5 (**p < 0.01). With regard to thermolysin, the reaction was significantly diminished at pH 8.0 when compared to all other pH conditions (****p < 0.0001). For neutral protease, there was significant difference between pH 7.0 and pH 8.0 (*p < 0.05). The assay was conducted using a substrate concentration of 20 μM (n = 2).

We also tested the influence of pH on enzyme activity. The results showed that all enzyme activities were influenced by pH except collagenase I (Fig. 5). For collagenase II, the reaction was significantly inhibited at pH 8.0 (RFU values 39250 ± 250), as compared with pH 6.8 (42750 ± 950) and pH 7.5 (43600 ± 400) (p < 0.01). With regards to thermolysin, the reaction was significantly diminished at pH 8.0 (17400 ± 200), when compared to all other pH conditions (pH 6.8, 26500 ± 200; pH 7.0, 27750 ± 250; pH 7.5, 25200 ± 800) (p < 0.0001). For neutral protease, there was a significant difference between pH 7.0 (23400 ± 300) and pH 8.0 (20000 ± 400) (p < 0.05).

### Effect of pancreatic proteases on the peptide substrate

**Figure 6:**
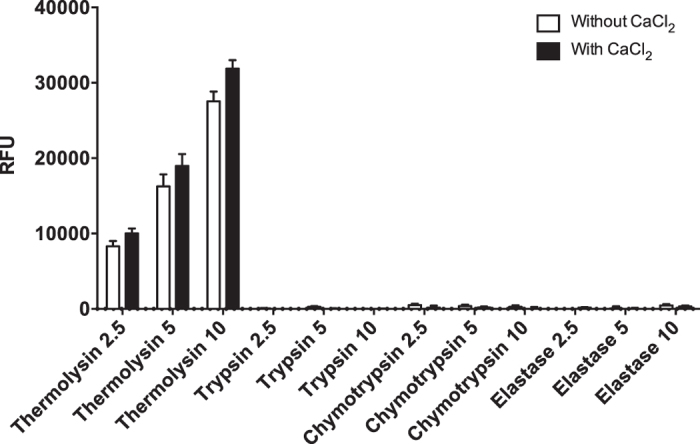
Reaction of endogenous pancreatic proteases and peptide substrate. Thermolysin demonstrated a dose dependent cleavage of peptide substrate both in the presence and absence of 4.14 mM CaCl_2_. All three pancreatic proteases: trypsin, chymotrypsin, and elastase showed no activity compared to thermolysin (2.5, 5, 10 μM), regardless of the presence or absence of CaCl_2_ (****p < 0.0001). The peptide substrate was used at a concentration of 50 μM (n = 3).

All the enzymes (collagenase I, collagenase II, Liberase MTF C/T, collagenase NB1, thermolysin, and neutral protease) cleaved the peptide substrate. In this study, thermolysin was used to compare with the pancreatic endogenous proteases. The results showed that thermolysin demonstrated a dose-dependent cleavage of peptide substrate both in the presence and absence of 4.14 mM CaCl_2._ The RFU values in the presence of CaCl_2_ are: 10016 ± 671 (2.5 μM), 18966 ± 1571 (5 μM), and 31883 ± 1134 (10 μM); in the absence of CaCl_2_ are: 8305 ± 707 (2.5 μM), 16255 ± 1602 (5 μM), and 27538 ± 1294 (10 μM). However, all three pancreatic proteases (trypsin, chymotrypsin, and elastase) showed no effect on peptide substrate, compared to thermolysin at 2.5, 5, 10 μM, regardless of the presence or absence of CaCl_2_ (p < 0.0001, Fig. 6).

## Discussion

We describe a novel peptide substrate for measuring enzyme activities of collagenase I, II, Liberase MTF C/T, collagenase NB1, thermolysin, and neutral protease. Our substrate is unique since it cannot be cleaved by pancreatic endogenous proteases such as trypsin, chymotrypsin, and elastase. It is highly specific for bacterial enzymes, sensitive, simple, and fast to monitor enzyme activities. Currently, there is no standard single assay to measure activities of all enzymes available to digest the pancreas and to free the islets. Although the Wünsch assay has contributed tremendously to the development of collagenase, it can only measure collagenase class II. It cannot be used for measuring collagenase class I, thermolysin or neutral protease^9^,^16^,^24^,^25^,^26^,^27^,^28^. On the other hand, our peptide substrate can be used to measure the activities of these enzymes including thermolysin and neutral protease. Roche Liberase MTF C/T and Serva collagenase NB1/neutral protease are GMP products and currently being used for human islet isolation. In this study, we compared Liberase MTF collagenase and Serva collagenase NB1. It was clearly showed that enzyme activities from various manufacturers are variable as shown in unit per ml, indicating that these enzymes though produced from *Clostridium histolyticum*, the strain variable might influence the product and stability. Indeed, Liberase MTF collagenase and Serva collagenase NB1enzyme activities were different as shown in Table 3. The enzyme activities were calculated using the modification of previously described formulation^29^.

Adult human pancreata have strong collagen bands, which can be degraded by Liberase MTF C/T Roche enzymes^11^. However, enzyme digestion of pancreata from younger donors is challenging because islets are not free from acinar cells resulted into embedded/mantled islets^10^,^11^,^30^,^31^. Therefore, Serva collagenase NB1 and neutral protease were used for pancreas digestion to free the islets from younger donors. Furthermore, with the known sequence of the peptide substrate, analysis of the cleavage sites on peptide substrate upon enzymatic reaction became possible. Therefore, characterization of ECM of the pancreata from different age groups may help to develop a cocktail of multiple enzyme(s) using our peptide substrate, overcoming the variability among age groups. Interestingly, our peptide substrate (A G G P L G P P G P G G) has 80–90% similar structure of collagen bands: I, II, III, IV, and V as shown in Table 1, using BLAST and the amino acid sequences of collagens as described in the results section. Islet mass and quality are current index for determining enzyme efficacy. Thus, our peptide substrate may replace current expensive and challenging procedure for pancreas digestion to free the islets. It would be of immense benefit to evaluate multiple enzymes using this peptide substrate so that appropriate enzyme cocktails could be prepared for tissue dissociation with minimal cell death^24^,^32^.

Interestingly, the cleavage sites of our peptide substrate by different enzymes that currently being used by multiple centers showed that Liberase MTF C/T was quite different from Serva collagenase NB1 (Fig. 1). It is well known that collagenase is Ca^2+^ dependent^11^. In this study, we performed enzyme kinetic study and used two important kinetic parameters V_max_ and K_m_ to compare the enzyme activity in the presence and absence of CaCl_2_. V_max_ reflects how fast the enzyme can catalyze the reaction. In this specific study, the higher V_max_, the faster the enzymes cleave the peptide substrate. Our data clearly showed that only collagenase I and neutral protease, the cleavage of the peptide was enhanced significantly in the presence of CaCl_2_ reflected by significantly higher V_max_ values, underlying that CaCl_2_ should always be used regularly during the digestion process for islet isolation. The results also showed that the enzymatic cleavage was significantly inhibited or diminished in the presence of zinc. Previous studies have shown that collagenase enzymes bind to islets post culture and attributed this as the cause of the deteriorating effects resulting in islet loss^33^. However, collagenase activity was undetectable in culture media taken from islet samples post culture using our highly sensitive peptide substrate. The pH value of the reaction buffer affects enzyme activity. Our results indicated that the enzyme activity was not affected at pH 6.8, pH 7.0, and pH 7.5. Thus, it is suggesting that the pH should be kept within the physiological range during digestion process for optimum islet isolation, cell function and survival post transplantation. Within the four sets of pH conditions in this experiment, only the pH 8.0 diminished enzyme activity, therefore the pH 8.0 condition is not recommend to perform enzyme assay when using our peptide substrate.

There are multiple endogenous pancreatic proteases such as trypsin, chymotrypsin, and elastase, which play an important role in hydrolysis of complex nutrients^34^,^35^. These proteases could be activated during the digestion process and thus may influence islet quality and yields^36^,^37^,^38^. However, our results showed that our peptide substrate cannot be cleaved by the three predominantly endogenous pancreatic proteases using similar conditions for evaluating microbial enzymes.

In conclusion, we synthesized a novel peptide that can be utilized for quantifying bacterial enzyme activity using a FRET assay. The peptide is microbial collagenase, thermolysin/neutral protease specific, and could not be degraded by endogenous pancreatic proteases such as trypsin, chymotrypsin, and elastase. The method can be applied to measure enzyme activities of the products from various sources. Furthermore, this method can be used to monitor collagenase activity and examine the effects that any additional activator or inhibitor may have on enzymatic function, particularly during the tissue digestion process.

## Methods

### Chemicals, Reagents, and Enzymes

Chemicals, including trypsin, chymotrypsim, elastase, 4 (dimethylaminoazo)benzene-4-carboxylic acid (dabcyl), 5(6)-carboxyfluorescein, N,N,N′,N′-Tetrakis(2-pyridinylmethyl)-1,2-ethanediamine (TPEN), ZnCl_2_, ZnSO_4_, NaSO_4,_ triethanolamine (TEA), ethylenediaminetetraacetic (EDTA), ethylene glycol-bis (β-aminoethyl ether)-N,N,N′,N′-tetraacetic acid (EGTA), and CaCl_2_ were purchased from Sigma-Aldrich (Saint Louis, MO). Highly purified collagenase class I, II and thermolysin samples were kindly provided by Roche Diagnostics (Roche Diagnostics, Roche Applied Science, Indianapolis, IN, USA). Collagenase NB1 and neutral protease were purchased from Serva (SERVA Electrophoresis GmbH, Heidelberg, Germany).

### Synthesis of Peptide Substrate

Peptide synthesis was designed and performed according to the general methods outlined in Kaplan *et al*.^39^ with some modifications using Fmoc-Lys (dde)-OH and K (dabcyl). After removal of the terminal Fmoc group, 5(6)-carboxyfluorescein was activated. The excess reagents were washed out with piperidine: DMF (1:4). The Lys (Dde) was deprotected using 2% hydrazine in DMF, washed with DMF and DCM and 4-dimethylaminoazobenzene-4′-carboxylic acid (dabcyl) was activated in the usual manner and coupled as any standard amino acid. The resin was washed and the peptide was cleaved from the resin by standard methods. The peptide was purified by HPLC as described previously^39^ and the sequence was confirmed by mass spectrometry. The stock peptide substrate was dissolved in DMSO and stored at −80 °C (light protected) until used.

### FRET Assay for Enzymatic Reaction

The reaction kinetics between enzymes and newly synthesized peptide substrate were evaluated using FRET assay. In brief, fluorogenic peptide substrate was synthesized in which donor and quencher (dabcyl) molecules were attached to corresponding amino acids. After cleaving by the enzymes, fluorophore quenching was diminished due to the separation of donor and quencher moieties^40^,^41^,^42^. As a result, the donor fluorescence increases dramatically, which can be measured with excitation 485 nm and emission at 535 nm^43^. The enzymatic reaction was performed in a 96-well round bottom black plate (Costar ID#3915, Corning, NY). Briefly, 190 μl of substrate with a final concentration of 5–80 μM and 10 μl of specific enzyme samples were added to each well, creating a final volume of 200 μl/well. A blank sample was used containing only substrate with no enzyme. In addition, enzyme samples were boiled for 5 min at 80 °C and used as a control. The plate was incubated (light protected) for 1 hr at 22 °C and the reaction was stopped by adding 50 μl of 40 mM EDTA solution (pH 8.0). The fluorescence was read at excitation 485 nm and emission 535 nm using the Tecan Magellan V 6.5 Genios plate reader (Tecan Systems, Inc., San Jose, CA, USA). Microsoft excel was used to further extrapolate the data.

### Enzyme Kinetic Assay

The Michaelis–Menten Model of enzyme kinetics and Lineweaver-Burk plot and equation were used to establish the rate of the enzymatic reaction in relationship to the substrate using known substrate concentrations^43^. The Michaelis–Menten model was chosen for analysis as it allows for the comparison of different enzyme enhancers and the effect of these activators on the enzymatic reaction^44^. The V_max_ and K_m_ values were calculated using GraphPad Prism. The substrate concentrations used in this kinetics study were: 5, 10, 20, 40, 60 and 80 μM. The concentrations of the tested enzyme were: 0.45 μg/ml for collagenase I, 0.3 μg/ml for collagenase II, 17 μg/ml for thermolysin, and 20 μg/ml for neutral protease. In this study, using the newly synthesized peptide substrates and enzymes, we tested following influential factors on enzyme activity: (i) effect of CaCl_2_ (4.14 mM) on enzymatic kinetics; (ii) effect of ZnSO_4_ (16.7 μM), ZnCl_2_ (16.7 μM), NaSO_4_ (16.7 μM); (iii) effect of chelating agents EGTA (25, 50, 100 mM); (iv) effect of pH (pH 6.0–8.0); (v) scaling-up concentrations (2.5, 5, 10 μg/ml) of pancreatic endogenous proteases (trypsin, chymotrypsin and elastase) were also used to examine the efficiency to cleave peptide substrate.

### Statistical Analysis

GraphPad Prism (GraphPad Software 6.0, La Jolla, CA, USA) was used for analyzing the data and generating the graphs. Both non-linear Michaelis-Menten and linear Lineweaver-Burk plots were used for the enzyme kinetics study. Kinetic parameters V_max_ and K_m_ were obtained from the Michaelis-Menten model by plotting the reaction velocity at different concentration of peptide substrate. Non-linear Michaelis-Menten curves for the enzymatic reaction in the presence and absence of CaCl_2_ were compared using Prism. All samples were run in duplicates, and results were reported as average ± standard error of the mean (SEM). One-way or two-way ANOVA analysis followed by Tukey’s multiple comparisons test was used to conduct multiple variable comparisons when applicable. Differences in data were considered significant when P values were less than 0.05. Coefficient of Variation (CV) was calculated using Microsoft Office Excel 2011.

## Additional Information

**How to cite this article:** Al-Abdullah, I. H. *et al*. Fluorogenic Peptide Substrate for Quantification of Bacterial Enzyme Activities. *Sci. Rep.* **7**, 44321; doi: 10.1038/srep44321 (2017).

**Publisher's note:** Springer Nature remains neutral with regard to jurisdictional claims in published maps and institutional affiliations.

## Supplementary Material

Supplementary Information

## Figures and Tables

**Table 1:** Comparison of amino-acid sequences of peptide substrate and human collagen types using BLAST.

	Max score	Total score	Query cover	E value	Identity	Accession
Peptide vs. Collagen type 1	25.7	1639	100%	5.00E-05	80%	Query_100135
Peptide vs. Collagen type II	25.7	1419	100%	5.00E-05	80%	Query_245089
Peptide vs. Collagen type III	25.7	1373	100%	5.00E-05	80%	Query_258809
Peptide vs. Collagen type IV	25.7	1252	100%	5.00E-05	90%	Query_120653
Peptide vs. Collagen type V	24.4	1831	100%	1.00E-04	80%	Query_26301
Peptide vs. Laminin	9.1	9.1	16%	61	100%	Query_229729
Peptide vs. Fibronectin	15.5	39.7	66%	0.25	80%	Query_19393

BLAST, Basic local alignment search tool.

Query sequence of peptide substrate: AGGPLGPPGPGG.

Complete sequences of Collagens, Laminin, and Fibronectin were obtained in FASTA format from UniProtKB (http://www.uniprot.org).

**Table 2:** Kinetic parameters of enzymes.

Enzymes	Vmax (RFU/min/μg)	Km (μM)
Collagenase I	808.8 ± 59.5	24.7 ± 4.8
Collagenase I + CaCl_2_	1845.0 ± 226.0	36.7 ± 10.2
Collagenase II	2676.0 ± 149.6	14.9 ± 2.7
Collagenase II + CaCl_2_	2802.0 ± 276	19.9 ± 5.6
Thermolysin	21.3 ± 0.6	24.1 ± 1.7
Thermolysin + CaCl_2_	28.2 ± 9.3	29.2 ± 23.8
Neutral Protease	18.8 ± 3.9	65.6 ± 24.5
Neutral Protease + CaCl_2_	30.3 ± 11.0	46.0 ± 34.9

RFU, relative fluorescent unit.

The values were expressed as Mean ± SEM.

**Table 3:** Enzyme activities from different lot of Liberase MTF collagenase and Serva collagenase NB1 using peptide substrate.

Enzymes	From manufacturer CoA		Calculated using the equation*	
	Activity units (U/ml)	Inter-lot CV (%)	Activity units using the peptide substrate (U/ml)	Inter-lot CV (%)
Liberase MTF collagenase				
Lot #1 L	142.95	3.58	10636292.10	5.42
Lot #2 L	142.8		11794945.83	
Lot #3 L	153.65		12075835.93	
Lot #4 L	144.1		11464940.96	
Average ± SD	145.87 ± 5.21		11493003 ± 623325	
Serva collagenase NB1				
Lot #1S	129.7	9.24	8022707.00	12.44
Lot #2S	143.15		10487038.69	
Lot #3S	118.5		10019338.78	
Lot #4S	118.3		8630865.76	
Average ± SD	127.41 ± 11.76		9289988 ± 1155469	

CoA, certificate of analysis; CV, coefficient of variation; *
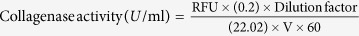

Where: RFU = Relative fluoresecent units.

0.2 = Reaction volume (ml).

22.02 = Milimolar extinction coefficient of substrate.

V = enzyme volume (ml).

60 = Total time of enzyme assay (min).

## References

[b1] Brennan D. C. . Long-Term Follow-Up of the Edmonton Protocol of Islet Transplantation in the United States. Am. J. Transplant., doi: 10.1111/ajt.13458 (2015). 26433206

[b2] Orr C. . Quantifying Insulin Therapy Requirements to Preserve Islet Graft Function Following Islet Transplantation. Cell Transplant. 25, 83–95, doi: 10.3727/096368915X687958 (2016). 25853639

[b3] Qi M. . Five-year follow-up of patients with type 1 diabetes transplanted with allogeneic islets: the UIC experience. Acta Diabetol. 51, 833–843, doi: 10.1007/s00592-014-0627-6 (2014). 25034311 PMC4801517

[b4] Balamurugan A. N. . Islet product characteristics and factors related to successful human islet transplantation from the Collaborative Islet Transplant Registry (CITR) 1999–2010. Am. J. Transplant. 14, 2595–2606, doi: 10.1111/ajt.12872 (2014). 25278159 PMC4282081

[b5] Johnson P. R., White S. A. & London N. J. Collagenase and human islet isolation. Cell Transplant. 5, 437–452 (1996). 8800512 10.1177/096368979600500403

[b6] Uscanga L., Kennedy R. H., Stocker S., Grimaud J. A. & Sarles H. Immunolocalization of collagen types, laminin and fibronectin in the normal human pancreas. Digestion 30, 158–164 (1984). 6389236 10.1159/000199100

[b7] Kennedy R. H. . Pancreatic extracellular matrix alterations in chronic pancreatitis. Pancreas 2, 61–72 (1987). 3575315 10.1097/00006676-198701000-00010

[b8] Cross S. E. . Key Matrix Proteins Within the Pancreatic Islet Basement Membrane Are Differentially Digested During Human Islet Isolation. Am. J. Transplant., doi: 10.1111/ajt.13975 (2016). 27456745

[b9] McCarthy R. C. . Development and characterization of a collagen degradation assay to assess purified collagenase used in islet isolation. Transplant. Proc. 40, 339–342, doi: 10.1016/j.transproceed.2008.01.041 (2008). 18374061

[b10] Szot G. L. . Successful clinical islet isolation using a GMP-manufactured collagenase and neutral protease. Transplantation 88, 753–756 (2009). 19920770 10.1097/TP.0b013e3181b443ae PMC2782539

[b11] Qi M. . The Choice of Enzyme for Human Pancreas Digestion is a Critical Factor for Increasing the Success of Islet Isolation. Transplant Direct 1, doi: 10.1097/TXD.0000000000000522 (2015). PMC4486320 26146662

[b12] Bertuzzi F. . Collagenase isoforms for pancreas digestion. Cell Transplant. 18, 203–206 (2009). 19499708 10.3727/096368909788341270

[b13] Linetsky E. . Improved human islet isolation using a new enzyme blend, liberase. Diabetes 46, 1120–1123 (1997). 9200645 10.2337/diab.46.7.1120

[b14] Nano R. . Islet isolation for allotransplantation: variables associated with successful islet yield and graft function. Diabetologia 48, 906–912, doi: 10.1007/s00125-005-1725-3 (2005). 15830183

[b15] Kin T., O’Gorman D., Senior P. & Shapiro A. M. Experience of islet isolation without neutral protease supplementation. Islets 2, 278–282 (2010). 21099325 10.4161/isl.2.5.12602 PMC3230559

[b16] Wuensch E. & Heidrich H. G. [on the Quantitative Determination of Collagenase]. Hoppe Seylers Z. Physiol. Chem. 333, 149–151 (1963). 14058277 10.1515/bchm2.1963.333.1.149

[b17] Tokmina-Roszyk M., Tokmina-Roszyk D., Bhowmick M. & Fields G. B. Development of a Forster resonance energy transfer assay for monitoring bacterial collagenase triple-helical peptidase activity. Anal. Biochem. 453, 61–69, doi: 10.1016/j.ab.2014.02.024 (2014). 24608089 PMC4260936

[b18] Saikumari Y. K. & Balaram P. An internally quenched fluorescent substrate for collagenase. Biopolymers 90, 131–137, doi: 10.1002/bip.20952 (2008). 18260138

[b19] Salamone M. . A new method to value efficiency of enzyme blends for pancreatic tissue digestion. Transplant. Proc. 42, 2043–2048, doi: 10.1016/j.transproceed.2010.05.107 (2010). 20692403

[b20] Baici A., Cohen G., Fehr K. & Boni A. A handy assay for collagenase using reconstituted fluorescein-labeled collagen fibrils. Anal. Biochem. 108, 230–232 (1980). 6257140 10.1016/0003-2697(80)90574-6

[b21] Lauer-Fields J. L. . Kinetic analysis of matrix metalloproteinase activity using fluorogenic triple-helical substrates. Biochemistry 40, 5795–5803 (2001). 11341845 10.1021/bi0101190

[b22] UniProt Knowledgebase < http://www.uniprot.org/>.

[b23] Standard Protein BLAST < http://blast.ncbi.nlm.nih.gov/Blast.cgi?PAGE=Proteins>.

[b24] Balamurugan A. N. . Identifying Effective Enzyme Activity Targets for Recombinant Class I and Class II Collagenase for Successful Human Islet Isolation. Transplant Direct 2, e54, doi: 10.1097/TXD.0000000000000563 (2016). 27500247 PMC4946501

[b25] Breite A. G., McCarthy R. C. & Dwulet F. E. Characterization and functional assessment of Clostridium histolyticum class I (C1) collagenases and the synergistic degradation of native collagen in enzyme mixtures containing class II (C2) collagenase. Transplant. Proc. 43, 3171–3175, doi: 10.1016/j.transproceed.2011.09.059 (2011). 22099748

[b26] Breite A. G., Dwulet F. E. & McCarthy R. C. Tissue dissociation enzyme neutral protease assessment. Transplant. Proc. 42, 2052–2054, doi: 10.1016/j.transproceed.2010.05.118 (2010). 20692405 PMC3031458

[b27] Marguerre A. K. & Kramer R. Lanthanide-based fluorogenic peptide substrate for the highly sensitive detection of thermolysin. Bioorg. Med. Chem. Lett. 19, 5757–5759, doi: 10.1016/j.bmcl.2009.07.152 (2009). 19720529

[b28] Voss E. W. Jr., Workman C. J. & Mummert M. E. Detection of protease activity using a fluorescence-enhancement globular substrate. Biotechniques 20, 286–291 (1996). 8825159 10.2144/96202rr06

[b29] Collagenase activities units < http://www.sigmaaldrich.com/catalog/product/roche/colldro?lang=en&region=US - x00AE;ion=US>.

[b30] Balamurugan A. N. . Suitability of human juvenile pancreatic islets for clinical use. Diabetologia 49, 1845–1854, doi: 10.1007/s00125-006-0318-0 (2006). 16783471

[b31] Meier R. P. . Islet of Langerhans isolation from pediatric and juvenile donor pancreases. Transpl. Int. 27, 949–955, doi: 10.1111/tri.12367 (2014). 24890668

[b32] Knapinska A. M. . Matrix metalloproteinases as reagents for cell isolation. Enzyme Microb. Technol. 93–94, 29–43, doi: 10.1016/j.enzmictec.2016.07.009 (2016). PMC5054980 27702483

[b33] Balamurugan A. N. . Harmful delayed effects of exogenous isolation enzymes on isolated human islets: relevance to clinical transplantation. Am. J. Transplant. 5, 2671–2681, doi: AJT1078 [pii]10.1111/j.1600-6143.2005.01078.x (2005). 16212626 10.1111/j.1600-6143.2005.01078.x

[b34] Whitcomb D. C. & Lowe M. E. Human pancreatic digestive enzymes. Dig. Dis. Sci. 52, 1–17, doi: 10.1007/s10620-006-9589-z (2007). 17205399

[b35] Simeone D. M. & Pandol S. J. The pancreas: biology, diseases, and therapy. Gastroenterology 144, 1163–1165, doi: 10.1053/j.gastro.2013.03.009 (2013). 23622124 PMC4919811

[b36] Tsukada M. . A model to evaluate toxic factors influencing islets during collagenase digestion: the role of serine protease inhibitor in the protection of islets. Cell Transplant. 21, 473–482, doi: 10.3727/096368911X605385 (2012). 22793055

[b37] Lakey J. R. . Serine-protease inhibition during islet isolation increases islet yield from human pancreases with prolonged ischemia. Transplantation 72, 565–570 (2001). 11544413 10.1097/00007890-200108270-00003

[b38] Nduaguibe C. C., Bentsi-Barnes K., Mullen Y., Kandeel F. & Al-Abdullah I. Serine protease inhibitors suppress pancreatic endogenous proteases and modulate bacterial neutral proteases. Islets 2, 200–206, doi: 10.4161/isl.2.3.11714 (2010). 21099314

[b39] Kaplan B. E., Hefta L. J., Blake R. C. 2nd, Swiderek K. M. & Shively J. E. Solid-phase synthesis and characterization of carcinoembryonic antigen (CEA) domains. J. Pept. Res. 52, 249–260 (1998). 9832303 10.1111/j.1399-3011.1998.tb01239.x

[b40] Weimer S., Oertel K. & Fuchsbauer H. L. A quenched fluorescent dipeptide for assaying dispase- and thermolysin-like proteases. Anal. Biochem. 352, 110–119 (2006). 16564490 10.1016/j.ab.2006.02.029

[b41] Cummings R. T. . A peptide-based fluorescence resonance energy transfer assay for Bacillus anthracis lethal factor protease. Proc. Natl. Acad. Sci. USA 99, 6603–6606, doi: 10.1073/pnas.062171599 (2002). 11997440 PMC124449

[b42] Matayoshi E. D., Wang G. T., Krafft G. A. & Erickson J. Novel fluorogenic substrates for assaying retroviral proteases by resonance energy transfer. Science 247, 954–958 (1990). 2106161 10.1126/science.2106161

[b43] Bagramyan K., Barash J. R., Arnon S. S. & Kalkum M. Attomolar detection of botulinum toxin type A in complex biological matrices. PLoS One 3, e2041, doi: 10.1371/journal.pone.0002041 (2008). 18446228 PMC2323579

[b44] Diaz S. A. . Probing the kinetics of quantum dot-based proteolytic sensors. Anal Bioanal Chem 407, 7307–7318, doi: 10.1007/s00216-015-8892-y (2015). 26215169

