# Supplementary material for: Fluorogenic Peptide Substrate for Quantification of Bacterial Enzyme Activities
Source: Sci Rep. 2017 Mar 13;7:44321. doi: 10.1038/srep44321 (PMC5347087; doi:10.1038/srep44321)
Supplement: Supplementary Information [file srep44321-s1.doc]

**Supplementary Information**

**Fluorogenic Peptide Substrate for Quantification of Bacterial Enzyme Activities**

**Ismail H. Al-Abdullah^1^, Karine Bagramyan^2^, Shiela Bilbao^1^, Meirigeng Qi^1^ and Markus Kalkum^2^**

1. Department of Translational Research and Cellular Therapeutics, Diabetes and Metabolism Research Institute, Beckman Research Institute of the City of Hope

2. Department of Molecular Immunology, Beckman Research Institute of the City of Hope

*Corresponding author:

Ismail H. Al-Abdullah

Department of Translational Research and Cellular Therapeutics, Diabetes and Metabolism Research Institute, Beckman Research Institute of the City of Hope

1500 E. Duarte Rd, Duarte, CA 91010

Tel: 626-256-4673 Ext 60109

Fax: 626-256-8704

Email: IAl-Abdullah@coh.org

**Methods**

**Mass spectrometry**

Collagenase peptide substrate was prepared as stock solutions (2.5 mM) in dimethyl sulfoxide (DMSO). A final substrate concentration of 10 μM was used for all enzymatic reactions, performed in a reaction buffer containing tris HCl (50 mM, pH 7.5) with NaCl (150 mM) and CaCl_2_ (10 mM). The samples were incubated at 37 °C for 10 to 180 min.

The digestion mixtures were diluted ten-fold with aqueous trifluoroacetic acid (TFA, 0.1%), desalted, concentrated using 10-µL-C18 ZipTip pipette tips (Millipore, Billerica, MA), and 1 μL of sample was spotted onto the MALDI target, mixed with half saturated α-cyano-4-hydroxycinnamic acid matrix (ProteoChem, Hurricane, UT), and left to dry. Mass spectrometric measurements were performed on a SimulTOF 200 Combo MALDI-TOF instrument (SimulTOF Systems / Virgin Instruments, Marlborough, MA), operating in reflectron positive ion mode. Three independent measurements were taken for each sample.


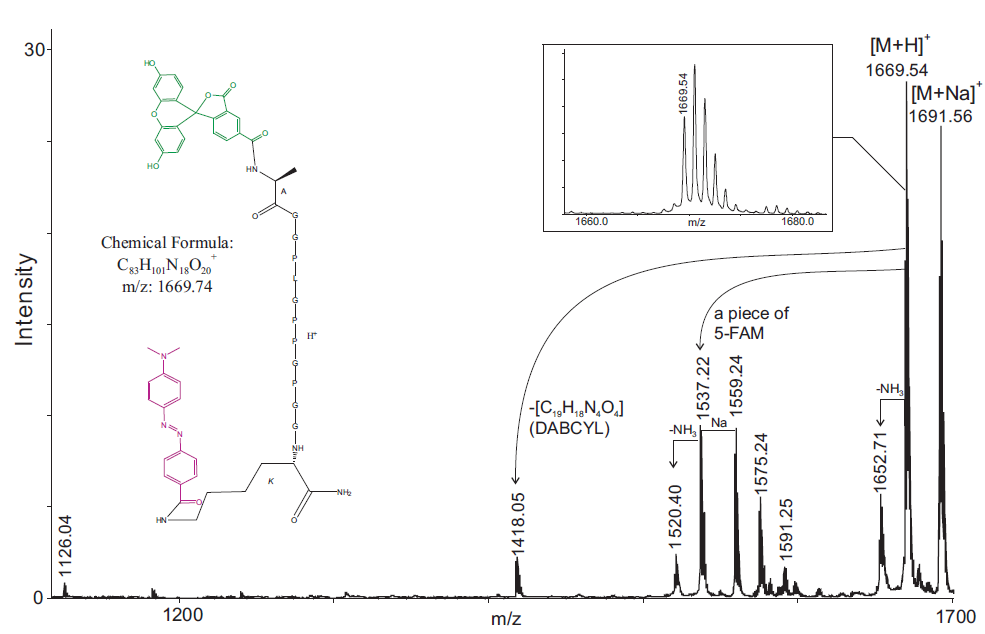


Figure S-1. Collagenase peptide MS spectrum obtained on a MALDI SimulTOF mass spectrometer. Inserts show the chemical formula of the collagenase peptide and a zoomed-in view of the spectrum of the parent ion at m/z 1669.54 corresponding to a theoretical molecular weight of the peptide. Arrows highlight the loss of ammonia, fragmentation of 5FAM, and DABCYL, as well as sodium adducts.

Table S-1. Summary report for collagenase substrate peptide fragment mass fingerprinting results obtained from the reaction of the peptide with different enzymes.

| 5Fam-AGGPLGPPGPGGK[DABCYL], 1669.54 Da | | | | | | |
| --- | --- | --- | --- | --- | --- | --- |
| Sequence of peptide cleavage products | Enzyme: | | | | | |
|  | Collage-nase,  class I | Collagenase, class II | Thermolysin | Neutral Protease  NB1 | Collagenase NB1 | Liberase MTF C/T |
|  | Peptide fragment peak from MALDI spectra | | | | | |
| AGGP (N-term.) |  |  | 658.19 | 658.19 |  |  |
| AGGPL (N-term.) | 771.28 | 771.28 | 771.28 | 771.28 |  | 771.28 |
| GPPGPGGK  (C-term.) |  |  | 916.47 | 916.47 |  |  |
| PPGPGGK(C-term.) |  |  |  |  |  |  |
| AGGPLGPP (N-term.) | 1022.40 | 1022.40 | 1022.40 | 1022.40 | 1022.40 | 1022.40 |
| PLGPPGPGGK  (C-term.) |  |  |  |  | 1125.61 |  |
| LGPPGPGGK (C-term.) |  |  | 1028.56 |  | 1028.56 |  |
| AGGPLGPPGPG  (N-term.) |  |  | 1233.50 |  |  |  |


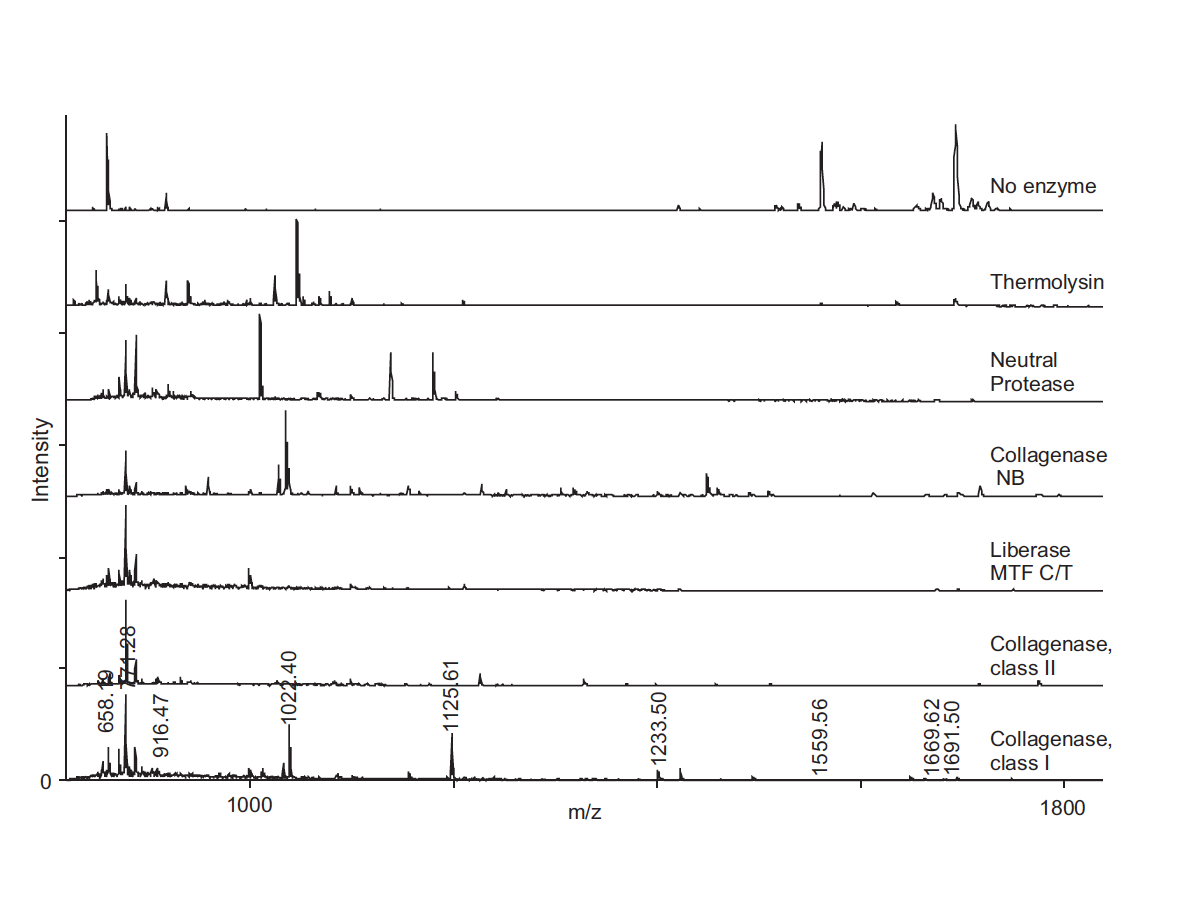


Figure S-2. Comparison of MALDI spectra of enzymatically digested substrate peptides. The spectrum of the undigested peptide substrate is in the top row.
